# Hepatic Glycogenosis: An Underdiagnosed Entity?

**DOI:** 10.7759/cureus.23853

**Published:** 2022-04-05

**Authors:** Sofia Garcês Soares, Renato Medas, Filipe Conceição, Roberto Silva, José Artur Paiva, Ana Cristina Carneiro

**Affiliations:** 1 Internal Medicine Department, Centro Hospitalar Tâmega E Sousa, Penafiel, PRT; 2 Gastroenterology and Hepatology Department, Centro Hospitalar Universitário São João, Porto, PRT; 3 Intensive Care Medicine Department, Centro Hospitalar Universitário São João, Porto, PRT; 4 Anatomic Pathology Department, Centro Hospitalar Universitário São João, Porto, PRT

**Keywords:** type 1 diabetes mellitus (t1dm), diabetic keto acidosis, liver biopsy, non-alcoholic fatty liver disease, hepatic glycogenosis

## Abstract

Hepatic glycogenosis (HG) is a rare complication of long-standing poorly controlled type 1 diabetes mellitus (T1DM), which is often misdiagnosed as non-alcoholic fatty liver disease (NAFLD). Despite the existence of several reports in the literature, it still is underrecognized, even among gastroenterologists. Differential diagnosis between these entities is essential since they have different prognoses.

We report a case of an 18-year-old female, with a medical history of poorly controlled T1DM, admitted to an intensive care unit with severe diabetic ketoacidosis (DKA). Upon admission, aminotransferases were significantly elevated; bilirubin and coagulation tests were normal. Despite adequate DKA treatment, she had persistently elevated aminotransferases and hyperlactacidemia. Imaging studies showed hepatomegaly and bright liver parenchyma. Extensive laboratory workup was negative for other causes of liver disease. So, a liver biopsy was performed, which was consistent with the diagnosis of HG. Under strict metabolic control, she had progressive improvement, achieving biochemical normalization within 6 months.

This case highlights the need for clinicians to be aware of this condition due to non-negligible differences between HG and NAFLD, with the latter progressing to fibrosis, and ultimately cirrhosis and hepatocarcinoma. On the opposite, HG is considered a benign condition, associated with an excellent prognosis that can be reversible after adequate metabolic control. Liver biopsy remains the gold standard method for HG diagnosis since it can distinguish it from NAFLD.

## Introduction

Hepatic glycogenosis (HG) is a rare complication of long-standing poorly controlled type 1 diabetes mellitus (T1DM) due to excessive intrahepatic glycogen accumulation. It was first described in 1930 by Pierre Mauriac in children with poorly controlled T1DM presenting with hepatomegaly with abnormal liver enzymes, poor growth, delayed puberty, cushingoid features, and hypercholesterolemia as the Mauriac Syndrome [1]. Later, several case reports described the presence of HG without the full spectrum of Mauriac Syndrome, both in adolescents and young adults with T1DM and less commonly with type 2 diabetes mellitus (T2DM) [2].

HG is still an underrecognized entity, even among gastroenterologists, and it is often misdiagnosed as non-alcoholic fatty liver disease (NAFLD) due to its similar presentation and difficult distinction based on clinical, biochemical, and radiological findings [3]. Differential diagnosis between these entities is important since they have different prognoses. NAFLD can progress to advanced fibrosis, liver cirrhosis, and hepatocarcinoma. In contrast, HG is considered a benign condition, without significant fibrosis and it can be reversible after adequate metabolic control [4]. Liver biopsy remains the gold standard test to make the differential diagnosis between HG and NAFLD, due to its unique pathologic features [5].

## Case presentation

We describe a case of an 18-year-old female, with a medical history of poorly controlled T1DM due to noncompliance with insulin therapy with 5 years of evolution. She presented in the emergency department with a 2-day history of upper abdominal pain, nausea, vomiting, and hyperglycemia (819 mg/dL). She denied fever, bowel transit changes, or consumption of hepatotoxins. She reported insulin pump malfunction over the last month.

On physical examination, she had no cushingoid facies and had a normal BMI (23.7 kg/m^2^). She was polypneic (respiratory rate 30), normotensive, tachycardic (heart rate 145 bpm), apyretic, and had a tender palpable liver. Laboratory workup revealed severe metabolic acidemia (pH 7.07, pO2 130.4 mmHg, pCO2 10.4 mmHg, HCO3 2.9 mEq/L, AG 29.3, lactates 4.16), leukocytosis and neutrophilia, cholestasis, transaminitis, and ketonuria. C-reactive protein (CRP), renal function, ionogram, bilirubin, and coagulation test were normal (Table 1). Abdominal ultrasound was positive for marked hepatomegaly (20 cm long axis) without focal lesions or biliary tract dilatation. Glycated hemoglobin (HbA1c) was 10.0%. So, the diagnosis of severe diabetic ketoacidosis (DKA) was made, and she was admitted to the intensive care unit (ICU) for prompt treatment with insulin perfusion.

**Table 1:** Laboratory diagnostic workup GOT: glutamate oxaloacetate transaminase; GPT: glutamic pyruvic transaminase; GGT: gamma-glutamyl transferase; ALP: alkaline phosphatase; LDH: lactate dehydrogenase; aPTT: activated partial thromboplastin time; PT: prothrombin time; HDL cholesterol: high-density lipoprotein cholesterol; calculated LDL cholesterol: calculated low-density cholesterol; TSH: thyroid-stimulating hormone; Free T4: free thyroxine; IgA: immunoglobulin A; IgM: immunoglobulin M; IgG: immunoglobulin G; CMV: cytomegalovirus; EBV: Epstein-Barr virus; HIV: human immunodeficiency virus; HBV: hepatitis B virus; HCV: hepatitis C virus

Parameter	Results	Reference value
Hemoglobin	14.5 g/dL	12.0–16.0
Leukocytes	25.62×10^9^/L	4.0–11.0
Neutrophils	75.2%	53.8–69.8
C- reactive protein	9.4 mg/L	<3.0
Platelets	582×10^9 ^/L	150–400
Urea	50 mg/dL	10–50
Creatinine	1.01 mg/dL	0.51–0.95
Sodium	133 mEq/L	135–147
Potassium	5.0 mEq/L	3.5–5.1
Chlorides	94 mEq/L	101–109
GOT	227 U/L	10–31
GPT	339 U/L	10–31
GGT	122 U/L	7–32
ALP	248 U/L	30–120
Total bilirubin	0.60 mg/dL	<1.20
LDH	418 U/L	135–225
Amylase	30 U/L	22–80
Lipase	5 U/L	7–60
Albumin	35 g/L	38.0–51.0
aPTT	21.6 seg	24.2–36.4
PT	12.6 seg	9.6–13.6
Fibrinogen	461 mg/dL	200 – 400
Ketonuria	60 mg/dL	<10
Total cholesterol	204 mg/dL	<200
HDL cholesterol	55 mg/dL	>60
Calculated LDL cholesterol	109 mg/dL	<130
Triglycerides	200 mg/dL	<150
Hb A1c	10%	4.0–6.0
TSH	1.6 UI/mL	0.35–5.00
Free T4	0.86 ng/dL	0.88–1.58
IgA	192 mg/dL	78–312
IgM	74 mg/dL	55–300
IgG	945 mg/dL	650–1500
Antinuclear antibodies	1/100	<1/100
Antimitochondrial antibodies	Negative	-
Anti-smooth muscle antibodies	Negative	-
CMV IgG antibody	134.6 AU/mL	<6.0
CMV IgM antibody	Negative	-
EBV VCA IgM antibody	Negative	-
EBV IgG antibody (Early)	<0.2 RU/mL	Negative < 0.9
EBV IgG antibody (EBNA)	>8.0 RU/mL	Uncertain 0.9–1.1
EBV IgG antibody (VCA)	>8.0 RU/mL	Positive >= 1.1
Heterophile antibodies	Negative	-
HIV 1 /2 antibodies	Negative	-
HBV antibodies	Negative	-
HCV antibodies	Negative	-
Ceruloplasmin	23.4 mg/dL	18.0–45.0
Alpha-1 antitrypsin	95.4 mg/dL	103.0–202.0

Despite the resolution of DKA in less than 24 hours upon admission, cholestasis, transaminitis, hyperlactacidemia (8.4 mmol/L), and abdominal pain persisted, which prompted a further workup.

**Figure 1:**
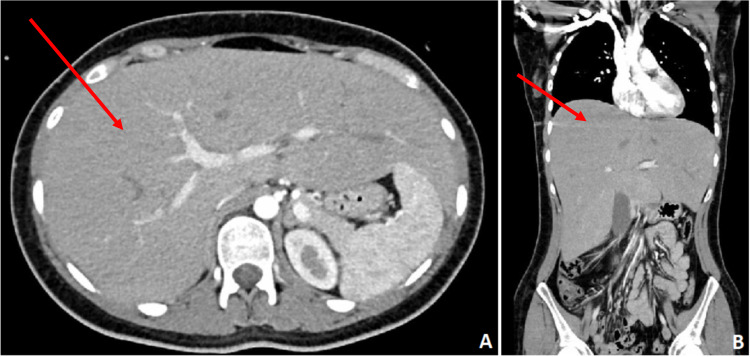
Cross (A) and coronal (B) sections of abdominopelvic computed tomography angiography revealing hepatomegaly

An abdominopelvic computed tomography angiography scan (Figure 1) confirmed hepatomegaly with bright parenchyma and revealed a hemorrhagic cyst in the left ovary (2.5 cm). A transvaginal ultrasound excluded an ovarian torsion. Workups for hepatitis A, B, C, and E viruses, cytomegalic (CMV) and Epstein-Barr viruses (EBV), human immunodeficiency virus (HIV), autoimmune hepatitis, alpha-1 antitrypsin deficiency, hemochromatosis, Wilson’s disease, and celiac disease were negative (Table 1).

**Figure 2:**
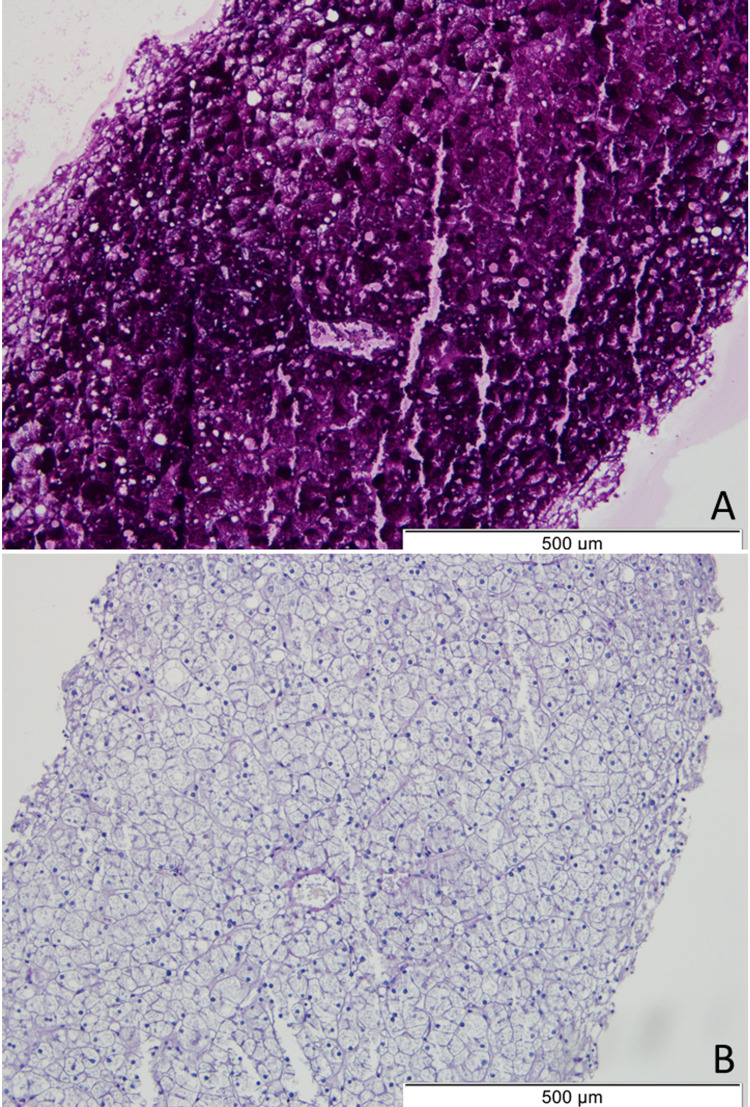
Liver histology Periodic acid–Schiff (PAS) stain showing large quantities of glycogen in the cytoplasm of hepatocytes (A). PAS with diastase (PAS-D) led to the digestion of cytoplasmatic glycogen, resulting in empty hepatocytes (“ghost cells”) (B), helping to differentiate glycogen from other PAS-positive elements in tissue samples.

**Figure 3:**
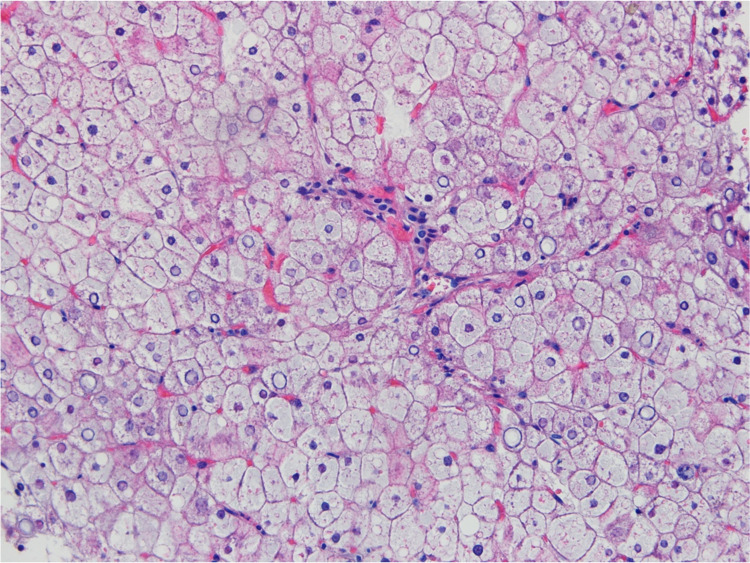
Liver histology Swollen and pale hepatocytes with nuclear glycogen pseudo-inclusions (hematoxylin and eosin (H&E)—200×).

A percutaneous liver biopsy was performed, which showed diffusely swollen hepatocytes with abundant and pale cytoplasm and thickened plasma membranes (paved appearance of liver parenchyma), multifocal nuclear glycogenization, and absence of fibrosis, consistent with the diagnosis of HG (Figures 2-3).

After three days, she was transferred from the ICU to the endocrinology ward. Under strict metabolic control, she showed progressive biochemical improvement, being discharged home six days later under a basal-bolus insulin regimen. She maintained a close follow-up and had an improvement in glycemic control (HbA1c 7.8%) and normalization of liver tests 6 months later.

## Discussion

The true incidence and prevalence of HG are unknown, but it is thought to be underestimated due to unawareness of this disease, even among gastroenterologists. One reason for that, it is the difficulty in its differential diagnosis with NAFLD, often leading to misdiagnosis [6]. The incidence of HG decreased significantly since the introduction of long-acting insulin and due to the growing recognition and awareness of glycemic control by diabetic patients and their caregivers, but it still exists [7].

Most reported cases of HG occurred in T1DM patients, while only ~2% were associated with T2DM [8]. In opposition, NAFLD is less common in T1DM, with an estimated prevalence lower than in the general population and it is 2-fold higher than in the general population in T2DM patients [9]. Other conditions were associated with HG such as dumping syndrome, anorexia nervosa, high-dose glucocorticoid use, and insulin overdose [8].

The exact mechanism responsible for HG is not fully understood, but wide fluctuations in glucose and insulin levels that can occur in diabetic patients with poor metabolic control may play an important role. The synthesis of hepatic glycogen is the consequence of the combination of high blood glucose levels (which promote the flow of glucose into hepatocytes) and hyperinsulinemia (which stimulates the conversion of glucose to glycogen) [10,11]. However, it is not understood why only some patients have the potential to develop HG.

Albeit initially described in children, several reports described this entity in adults (without the full spectrum of Mauriac Syndrome). So, HG can appear at any age, although most cases occur in adolescence, with a slight predominance in females (~62%) [8]. Clinical presentation of HG varies from an asymptomatic elevation of liver enzymes to hyperglycemia-related symptoms such as polyuria, polydipsia, weight loss, and lethargy, and other symptoms such as abdominal pain, nausea, vomiting, and anorexia. HG is often seen in patients with frequent episodes of DKA (abdominal pain, nausea, and vomiting) [8].

The most common finding on physical examination is hepatomegaly without splenomegaly, which is present in more than 90% of the reported cases. Rapid enlargement of the liver may cause visceral pain secondary to Glisson’s capsule stretching [8]. Rarely, ascites may be present due to sinusoidal compression by swollen hepatocytes [12]. Our patient presented only with tender hepatomegaly, which may reflect a rapid hepatic glycogen accumulation due to malfunction of the insulin pump and poor glycemic control. The occurrence of DKA was probably the trigger to develop elevation of liver enzymes.

Laboratory workup often presents mild to moderate elevation in liver aminotransferases, with a predominant elevation of glutamate oxaloacetate transaminase (GOT) over glutamic pyruvic transaminase (GPT) (ratio GOT/GPT >1). Most reported cases showed a hepatocellular pattern, although a mixed pattern or even a cholestatic pattern can rarely occur [13]. Marked elevations of aminotransferases (up to 100× the upper limit of normal) have been reported, generally in patients presenting with DKA. Dehydration in this setting may have a role in such elevation due to transient liver hypoperfusion [14]. Albeit rare, elevation of GPT can also occur as we have seen in our patient [13]. Liver function is usually preserved [15].

Our patient had persistent hyperlactacidemia despite adequate treatment of DKA. Persistently elevated lactic acid can be seen in patients with HG who present with DKA without hypoperfusion [16]. The exact mechanism is unclear. A reduction in gluconeogenesis in the liver may raise lactate levels in the body. Therefore, lactic acidosis in HG could be explained by reduced gluconeogenesis with inhibition of the conversion of pyruvate to glucose and shifting its metabolism to lactate [17].

Diagnosis can be difficult because laboratory and imaging tests are not pathognomonic [15]. It includes the exclusion of other causes of liver damage: infectious (hepatitis B virus (HBV), hepatitis A virus (HAV), and hepatitis C virus (HCV)), metabolic (Wilson disease, hemochromatosis), obstructive diseases, autoimmune diseases, and drugs [8]. It is essential to test for autoimmune antibodies (antinuclear antibodies (ANA), anti-smooth muscle, and antimitochondrial) as there is an association between T1DM and autoimmune hepatitis [8]. This patient had only slightly positive ANA. HG and glycogen storage disease (GSD) can present similarly, so differentiating between them by genetic testing is important considering they have wide differences in their management. However, GSD often presents in the neonatal period or early infancy [18].

Once excluded from the above causes, the main differential diagnosis is NAFLD which presentation can be similar to HG. Imaging studies may help to reach the correct diagnosis, however, they have several limitations. Abdominal ultrasound is not useful to distinguish them, since in both cases it shows hepatomegaly and increased echogenicity of liver parenchyma [19]. Sweetser suggested that a bright liver on a CT scan (compared to the spleen), without the administration of contrast, can help in the differential diagnosis. A hyperdense liver may be seen in HG, compared to a hypodense liver in NAFLD [20]. However, the difference may be subtle and only provides qualitative information. So, neither abdominal US nor CT scan is a useful imaging method for the definitive diagnosis of HG. Gradient dual-echo magnetic resonance imaging (MRI) has been reported to be helpful in differentiating HG from NAFLD. HG presents with hypointense or isointense on T2-weighted images and hyperintense on T1-weighted images [21]. In NAFLD, in-phase and out-of-phase gradient T1-weighted images demonstrate signal dropout on the out-of-phase image due to the presence of fat deposition in the liver [22]. If there is no significant difference in the signal intensities between the two phases, then the results are not consistent with intrahepatic fat storage and are more consistent with HG [21].

Due to the inaccuracy of non-invasive methods, liver biopsy remains the gold standard for HG diagnosis. The timing for performing the biopsy is not defined in the literature, but it is known that this is the only way to diagnose HG and it is the only way to distinguish it from NAFLD. The fact that this patient maintained, despite the resolution of the DKA, abdominal pain, cholestasis, transaminitis, and hyperlactacidemia, led to the fear of possible organ dysfunction, leading to an early biopsy. Hematoxylin and eosin (H&E) stain shows pale and swollen hepatocytes, thickened plasma membranes, increased cytoplasmic volume, and glycogenotic nuclei (empty nuclei with ring-like chromatin elements). A mosaic or paved appearance of liver parenchyma may be seen due to sinusoidal compression of swollen hepatocytes [14]. Periodic acid-Schiff (PAS) stain is positive as it stains glycogen. The addition of diastase will cause enzymatic breakdown of glycogen, leading to empty hepatocytes, also called “ghost cells” [15]. Typically, the architecture of liver parenchyma remains intact. Most cases show no or minimal portal inflammation, steatosis, or fibrosis. However, recent reports described the presence of variable degrees of fibrosis including bridging fibrosis [23]. The implication of these findings is not yet known and should be addressed in future research. Conversely, NAFLD may show macrovesicular steatosis, mild lobular and portal inflammation, and varying degrees of fibrosis. In the presence of hepatocellular injury and fibrosis, there is an increased risk of progression to steatohepatitis, cirrhosis, and hepatocarcinoma [24].

After diagnosis, improving glycemic control is the mainstay of treatment. In most cases, adequate metabolic control with intensive insulin regimens results in improvement and resolution of clinical and biochemical features in 2 to 14 weeks [3]. Since recurrence of HG may be seen in the context of repeated episodes of DKA, all patients may be under strict surveillance to ensure adequate glycemic control over time [25]. Reversal of HG has also been reported following pancreatic transplantation in people with diabetes [26]. This patient was discharged under insulin therapy with a basal-bolus regimen, considering her preference and lack of motivation to restart the insulin perfusion pump. Although the analytical reassessment took place 6 months after discharge, it does not mean that its resolution could not have been earlier.

## Conclusions

In conclusion, the overall prognosis of HG is excellent, being reversible after adequate metabolic control. The authors consider that is important to be aware of this condition since it has a simple and effective treatment. Given the huge differences in prognosis between HG and NAFLD, liver biopsy is mandatory to ascertain the diagnosis and, more importantly, to avoid misdiagnosis.

In addition, it is important to keep in mind that this is a possible cause of liver enzyme changes in patients with poorly controlled DM and that it often goes unnoticed.
